# Effect of Chemical Etching on the Supercapacitive Performance of Electroless Ni-B Coatings

**DOI:** 10.3390/ma18153544

**Published:** 2025-07-29

**Authors:** Mate Czagany, Gabor Meszaros, Daniel Koncz-Horvath, Adrienn Hlavacs, Mark Windisch, Byungil Hwang, Peter Baumli

**Affiliations:** 1 Institute of Physical Metallurgy, Metal Forming and Nanotechnology, University of Miskolc, Miskolc-Egyetemvaros, 3515 Miskolc, Hungary; 2 HUN-REN Natural Sciences Research Centre, 1117 Budapest, Hungary; 3 Department of Materials Physics, Eötvös Loránd University, 1117 Budapest, Hungary; 4 Bay Zoltán Nonprofit Ltd. for Applied Research, 1116 Budapest, Hungary; 5 School of Integrative Engineering, Chung-Ang University, Seoul 06974, Republic of Korea

**Keywords:** electroless Ni-B, acid etching, pseudocapacitance, supercapacitor, porous structure, cyclic life

## Abstract

In our study, supercapacitor electrodes were prepared by depositing electroless Ni-B coating on copper plates, followed by nitric acid etching. The composition and the micro- and phase structure of the coatings were investigated by ICP-OES, PFIB-SEM, and XRD techniques. The original pebble-like structure of the coating consists of 0.8–10 µm particles, with an X-ray amorphous phase structure. The surface morphology and porosity of the coating can be tuned simply by changing the etching time. The supercapacitive performance of the electrodes was evaluated by means of cyclic voltammetry, galvanostatic charge–discharge, and electrochemical impedance spectroscopy measurements. The capacitance of the coating was found to vary on the etching time according to a maximum function, allowing for the determination of an optimal duration to obtain a specific capacitance of 157 mF/cm^2^ (at 0.5 A/g). An excellent charge storage retention of 178% was found after 5000 CV cycles at a scan rate of 50 mV/s owing to the evolved electrochemically active network on the surface of the electrode, indicating a long-term stable and reliable electrode.

## 1. Introduction

Electroless Ni-B coatings are widely used in various fields of industry, e.g., aerospace, automotive, chemical, and electrical industries [1,2], due to their excellent mechanical and corrosion resistant properties [3,4,5]. The presence of boron (B) essentially affects the microstructure of the coating: at low B content, the coating is mostly nano/microcrystalline, while at higher B content, the amorphous phase becomes more dominant [6]. The atomic disorder of the coating promotes an increase in the number of chemically active sites on the surface, where electrochemical reactions (e.g., redox processes) can take place. Furthermore, low crystallinity provides more efficient diffusion channels for electrolytic ions [7]. This allows them to be used in other fields of application such as catalysis, microelectronics, and electrochemical energy storage [8,9,10].

Supercapacitors (SCs) are electrochemical energy storage devices that have received significant scientific attention in recent years as they can combine high power density with fast charge/discharge cycles, good energy storage capability, and long cycle life [11,12,13,14]. The electrode material has a great role in determining the charge storage capability; thus, most studies focus on new designs to improve electrochemical performance [15,16]. Electroless Ni-B coatings are particularly promising as they have good electrical conductivity, excellent mechanical properties, and corrosion resistance [17,18,19]. Furthermore, the production of these coatings is simple, cost-effective, and well scalable, which gives an additional industrial advantage.

When combined with alkaline electrolytes (e.g., KOH, LiOH), Ni-B and Ni-P coatings possess pseudocapacitive behavior [20,21,22]. The electrochemical properties of the coatings are highly dependent on their surface morphology, structure, and composition [21]. Several studies demonstrated that modification of the specific surface area (SSA), thus introduction of a porous structure into the electrode materials, can effectively enhance the charge storage capability [23,24,25,26,27]. Chemical etching is an easy way to increase the SSA of a metal-based coating. Acid etching was reported to be an effective method to increase the specific surface area of Ni-P coatings [28,29]. However, very few studies have been conducted on the supercapacitive behavior of Ni-B coatings, and no study has reported on the ex situ structural modification of the coating and its effect on electrochemical behavior.

In this research, the aim was to improve the supercapacitive performance of electroless Ni-B-coated Cu electrodes by introducing a porous structure via chemical etching in 7 M HNO_3_. The effect of etching is investigated on the compositional change, microstructure, and electrochemical behavior of electroless Ni-B coatings deposited on pure Cu plates. The electroless coating method combined with chemical etching offers a simple, cost-effective technique to prepare supercapacitor electrodes that can be flexibly integrated into industrial practice.

## 2. Experimental Section

### 2.1. Electrode Preparation

The SC electrodes were prepared by depositing electroless Ni-B coatings on the surface of Cu plates. The composition of Cu is listed in Table 1. Prior to deposition, the plates were cut to the size of 20 mm × 11 mm × 1 mm and ground with #500 and #800 SiC paper, followed by polishing (down to 1 µm). The surface of the plates was cleaned ultrasonically in acetone (for 3 min), followed by a surface activation process by immersing the plates in dilute H_2_SO_4_ solution (10 wt.%) for 30 s. Distilled water rinsing was applied after each step.

The composition of the electroless bath and the deposition parameters can be seen in Table 2, which were optimized in our previous research to produce Ni-B coatings with a high specific area and high capacitance [21]. Nickel chloride and ethylene diamine (EDA) were obtained from VWR Chemicals Ltd. (Radnor, PA, USA), NaOH was purchased from Scharlab Ltd. (Barcelona, Spain), NaBH_4_ was purchased from Sigma-Aldrich (St. Louis, MO, USA), and thiourea was obtained from Reanal Private Ltd. (Budapest, Hungary). Etching was performed by immersing the Ni-B-coated Cu electrodes in 7 M HNO_3_ solution for different time periods (5 s, 10 s, 15 s, 17 s, 20 s), followed by distilled water rinsing.

### 2.2. Characterization

The surface morphology and composition of the electroless coatings were studied using a Helios G4 PFIB CXe plasma-focused ion beam scanning electron microscope (PFIB-SEM) (Waltham, MA, USA) equipped with an EDAX Octane Elect EDS System (Mahwah, NJ, USA). The B content of the coatings was determined by a Varian 720 ES (Santa Clara, CA, USA) inductively coupled optical emission spectrometer (ICP-OES). Measurements and evaluations of the 3D morphology of the electrodes were performed using a TESCAN VEGA 4 scanning electron microscope (Brno, Czech Republic) and Digital Surf MountainsLab (Mountains^®^ 11) surface evaluation software. Images of the surfaces were taken at 10k magnification using a 4-segment backscattered electron detector. During image acquisition, separate images of the same area were captured using each of the four segments. These were then converted into a 3D image using the MountainsLab Advanced Topography module (Besançon, France). Prior to processing the final topographical data, surface plane correction was applied. The phase structure of the coatings was studied by a Bruker D8 Discover X-ray diffractometer (Karlsruhe, Germany) with Cu K-alpha radiation, 40 kV, and 40 mA generator settings. Measurements were recorded at a 0.007° (2 Th)/19.20 s speed.

Electrochemical investigation was performed using a three-electrode cell system, with Ni-B-coated Cu plates as the working electrode, Pt-coated platinum foil (7 mm × 7 mm) as the counter electrode, and Ag/AgCl/3MKCl (+0.210 vs. SHE) as the reference electrode. Cyclic voltammetry (CV) and galvanostatic charge–discharge (GCD) measurements were performed in 2 M KOH electrolyte using an Autolab PGSTAT302N potentiostat–galvanostat (Zeist, The Netherlands), controlled by NOVA 2.1 software. CV analysis was carried out at the potential interval of 0–0.5 V with scan rates between 10 and 100 mV/s, while GCD was performed at the same potential interval of 0–0.5 V. The specific charge storage (Q_S_) and specific capacitance (C_S_) were determined in units of the macroscopic surface area of the electrode, which were calculated from the results of CV and GCD using the following equations [21,29]:

(1) $$Q_{S,CV}=\;\frac{\int_{0}^{∆V}\left|I(V)\right|\;dV}{2\cdot v\cdot A}$$

(2) $$C_{S,GCD}=\frac{2\cdot I\cdot \int_{t_{Vmax}}^{t_{Vmin}}V\left(t\right)dt}{{\left(∆V\right)}^{2}\cdot A}$$

where $\int_{0}^{∆V}\left|I(V)\right|$ is the integrated area of a full cycle (anodic and cathodic processes) of a CV curve, νis the scan rate ($\frac{V}{s}$), ∆V is the potential range of one CV cycle (V), and A indicates the macroscopic surface area of the electrode. EIS measurements were carried out using a Zahner Electric IM6e (Neudrossenfeld, Germany) potentiostat in the 100 kHz–0.1 Hz frequency range with a 10 mV_RMS_ value. A cylindrical Pt net served as the counter electrode while a saturated calomel electrode supplemented with a platinum pin through a 1 mF capacitor was applied as the reference electrode. The working electrode was placed at the center of the platinum net cylinder. Fitting of the obtained impedance data was carried out with Zview v.40h software.

## 3. Preliminary Results

### 3.1. Composition and Microstructure

**Figure 1:**
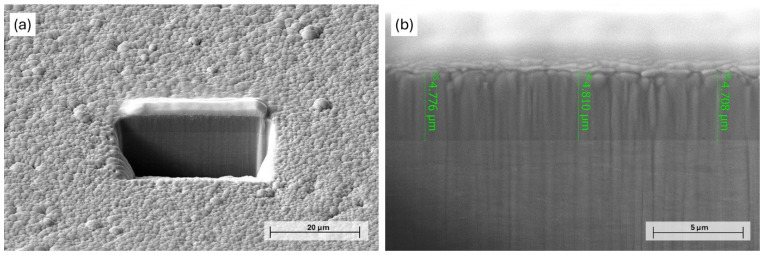
PFIB-SEM image of the (**a**) milled area on the surface of Ni-B coating and (**b**) cross-section of as-deposited Ni-B.

**Figure 2:**
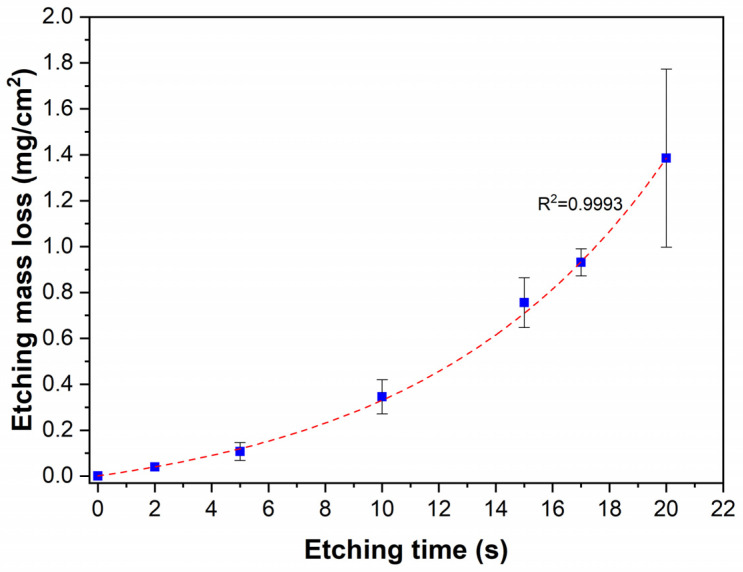
Etching mass loss of the Ni-B coatings as a function of etching time.

The effect of chemical etching of Ni-B coatings was studied by different approaches. The mass of the as-deposited coating was 22 ± 2 mg, with an average thickness of 4.8 µm (Figure 1) and a B content of ~6.61 wt%. The composition of the as-deposited Ni-B coating is listed in Table 3. The presence of C and O originates from surface contamination and residual organic compounds from the electroless bath, while O can refer to surface oxidation as well. The mass loss of the Ni-B-coated Cu samples can be seen in Figure 2 for different etching times. The dissolution of the coating can be characterized by an exponential curve, where the loss is below 10% in the first 12–13 s, referring to slow initial chemical reactions between the coating and the acidic solution. The dissolution then increases at a higher rate, resulting in a relatively high deviation in mass loss after 20 s, where the coating can be mechanically detached from the surface of Cu, thus giving inconsistent mass results.

The initial slow dissolution could be explained by the presence of a thin passive oxide (NiO, B_2_O_3_) layer on the coating surface. After dissolution, a more electrochemically active surface is exposed to the acidic medium. Próchniak et al. [30] observed that the dissolution of a relatively thick (110–120 µm) electrodeposited Ni coating in cc. HNO_3_ proceeded linearly in time over a few hours. The linear dissolution process was preceded by an initial slower dissolution with a quickly increasing rate. It is postulated that the exponential dissolution is limited to the investigated time interval, as the reaction products can accumulate in the boundary layer and the dissolved metal ions can slow down the dissolution process (ionic background effect), as was observed in the case of steel [31].

**Figure 3:**
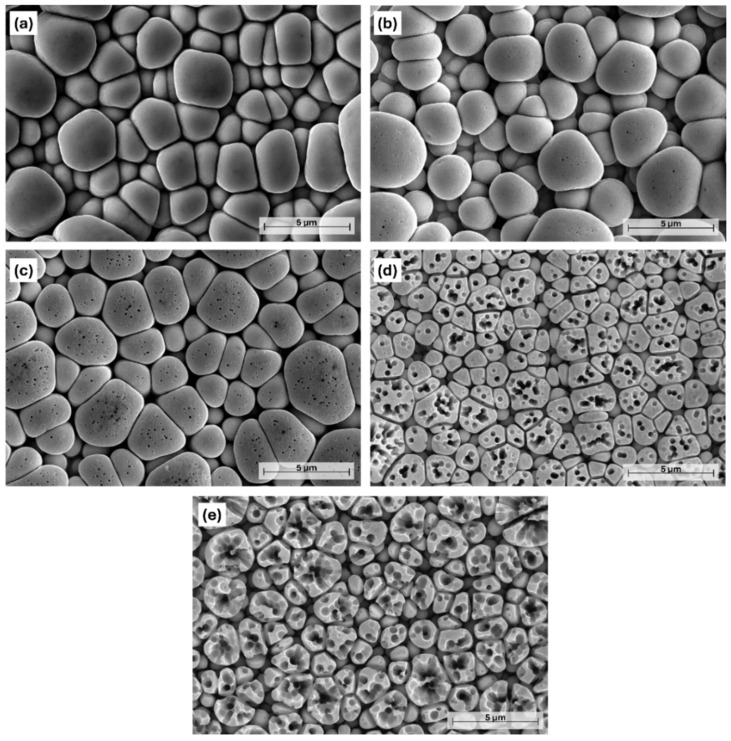
PFIB-SEM images of the Ni-B coatings with different etching time: (**a**) as-deposited Ni-B, (**b**) 5 s, (**c**) 10 s, (**d**) 15 s, (**e**) 17 s.

**Figure 4:**
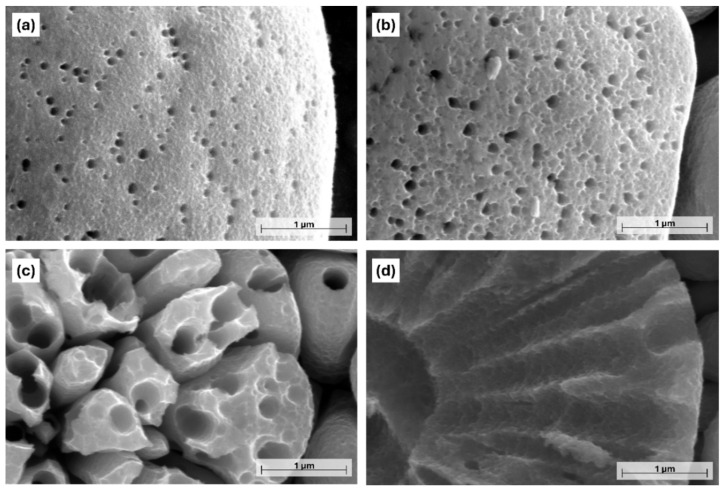
High-magnification PFIB-SEM images of the Ni-B coatings with different etching times: (**a**) 5 s, (**b**) 10 s, (**c**) 15 s, (**d**) 17 s.

From an optical point of view, the etching resulted in a darker tone to the originally gray color of the coating. The black shade can be primarily attributed to the formation of a rough porous surface structure and surface oxidation [32]. This is supported by Figure 3, showing the effect of acid etching on the microstructural change of the Ni-B coatings. The as-deposited coating has a nodular, pebble-like structure that is characteristic of electroless Ni-B coatings [33,34], with a nodule size ranging from 0.8 to 10 µm. These particles are separated by shallow irregular-shaped grooves (particle boundaries), which give the original pore structure of the coating [21]. After 5 s of etching, scattered submicron-sized pores appeared on the surface of the nodules, with a relatively regular circular shape and a diameter of 40–140 nm (Figure 3b and Figure 4a).

**Figure 5:**
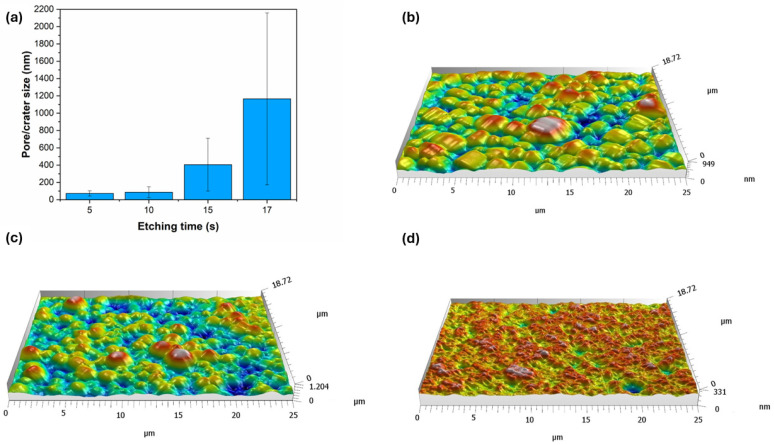
(**a**) Pore and crater size of Ni-B coatings as a function of etching time. Three-dimensional surface topography map of Ni-B coatings etched for (**b**) 0 s, (**c**) 5 s, (**d**) 15 s.

The location of the pores is random, with no specific arrangement. When the etching time was increased to 10 s, the originally smooth surface became ruffled owing to the appearance of numerous low-depth cavities (Figure 4b). At the same time, the porosity of the coating was increased as well; some of the neighboring deeper pores merged, thus forming pores with a larger diameter (140–250 nm). After 15 s, the size of the pores significantly increased, with an average size of 405 nm (Figure 5a). Their size distribution was wider, and their shape was mostly circular or elliptical with well-defined boundaries. Another feature of etching is the transformation of the pebble-like structure into an increasingly flat surface, and the widening of the boundaries separating the particles (Figure 3d). Seventeen seconds of etching resulted in the appearance of large micron-sized (Figure 5a) irregularly shaped craters in the structure of the coating (Figure 3e), while no pores could be found below 200 nm at this stage.

The morphology of the coatings was further studied by acquiring 3D topographies (Figure 5b–d). This method cannot be used to evaluate the fine interconnected pore structure, but it can provide insight into the microstructural changes during etching. The as-deposited coating (Figure 5b) shows a homogeneous, moderately structured topography, with ~950 nm peak-to-valley (PV) depth. This surface layer is accessible to electrolytic ions, thus serves as an effective surface area for the electrochemical processes. After 5 s of etching, the original particle size starts to decrease in parallel with the deepening of the particle boundaries (Figure 5c), resulting in a PV depth of ~1200 nm. This means that in addition to the pore structure, the widening–deepening boundaries (grooves) can also contribute to the increase in the specific surface area of the coating. Meanwhile, 15 s of etching gives the coating (Figure 5d) a highly porous, rich, and finely structured surface. A large number of irregularly shaped pores and cavities can be seen, with significant variation in depth, creating a well-defined three-dimensional structure. The etching, however, decreased the PV depth to ~331 nm, resulting in the flattening of the surface (Figure 3d).

**Figure 6:**
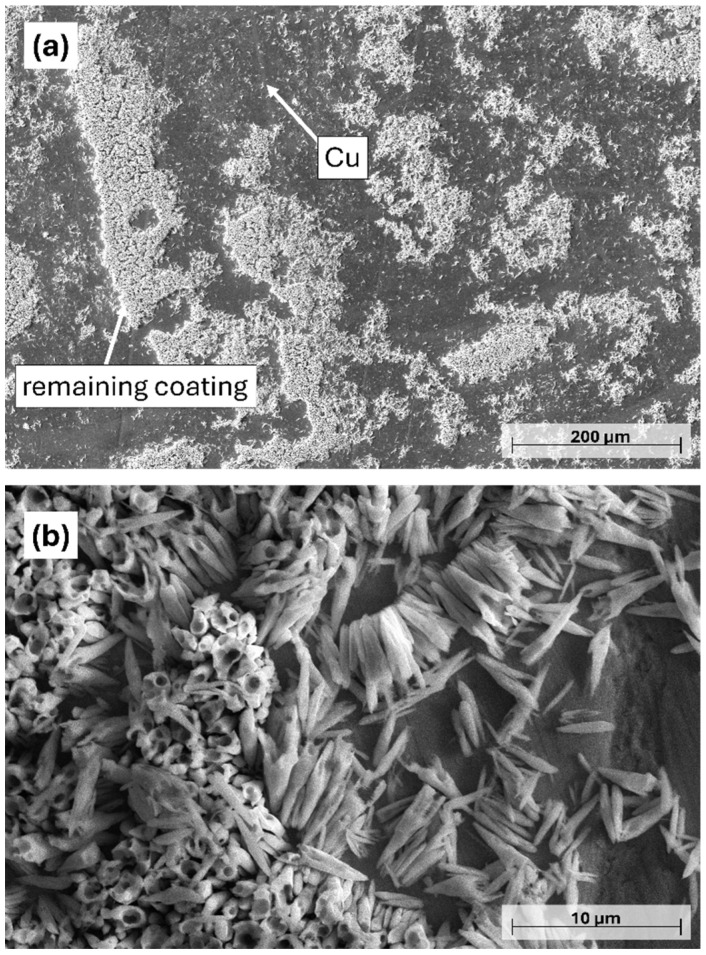
PFIB-SEM images of the Ni-B-coated Cu plates after 20 s of etching. (**a**) 250×, (**b**) 5000× magnification.

As expected from the mass loss after 20 s of etching (Figure 2), the coating was stripped from a high proportion of the Cu surface (Figure 6a). Furthermore, the remaining Ni-B coating transformed into cylindrical nickel structures with a length of 1.5–4.5 µm (Figure 6b). These morphological elements are remnants of the initial columnar structure of the coating that were selectively dissolved during etching, thus forming hollow/semi-hollow cylinders etched along the longitudinal axis of the columns. This phenomenon is in accordance with the previous findings, confirming that the atoms are not uniformly removed from the coating structure during the etching process. Compared to the etching of Ni-P coatings reported by Lin et al. [29] and Brown et al. [35], the etching mechanism is slightly different for Ni-B, as mostly nano-granular structures and stalagmite-like morphologies were formed in these cases, respectively. In our study, the transition from a pebble-like structure into cylindrical particles proceeds via surface ruffling, pore growth, and pore deepening.

### 3.2. Phase Structure

**Figure 7:**
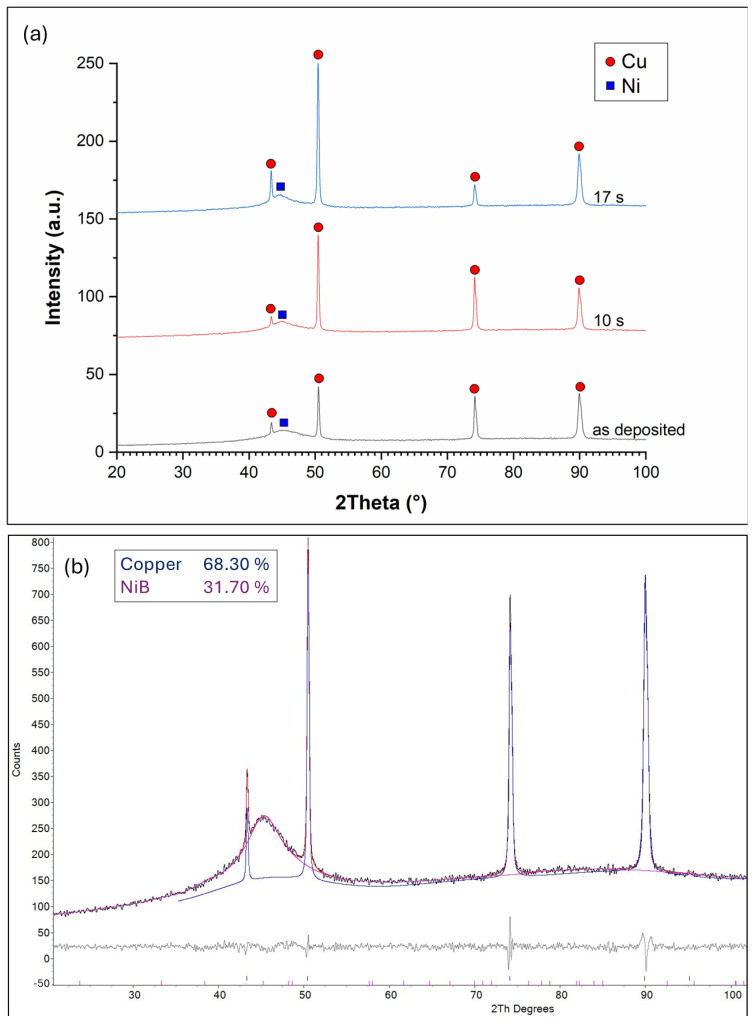
(**a**) XRD diffractograms of the Ni-B coatings etched for different time periods: as-deposited, 10 s, and 17 s; (**b**) Rietveld analysis of the diffractogram of as-deposited Ni-B.

The XRD diffractograms of the Ni-B coatings are shown in Figure 7a. The reflections of the Cu plate are clearly visible at 2Θ = 42.5°; 50.5°; 74.5°, and 90°, indicating its polycrystalline structure. The presence of Ni is only indicated near 2Θ = 44.5° where a broad peak can be observed, which typically refers to the X-ray amorphous structure of the Ni-B coatings. Ni-B coatings deposited by the electroless process are known to possess microcrystalline Ni and amorphous Ni-B phases, with increasing amorphous ratio as a function of B content [36,37]. To further study the phase structure, Rietveld analysis was applied (Figure 7b). The measured diffraction curve could only be fitted by assuming an amorphous or very finely crystalline Ni-B phase. The absence of crystalline Ni peaks suggests that the coating is largely amorphous, containing Ni-based domains (~2 nm, calculated by Scherrer equation) with short-term ordering, embedded in an amorphous Ni-B matrix, without forming a well-developed crystalline Ni or stoichiometric NiB phase. After 10 and 17 s of etching, the intensity of Cu reflection at 2Θ = 74.5° is increased, as well as the reflection at 2Θ = 42.5° after 17 s of etching, which is due to the reduction in the thickness of the Ni-B coating. No increase in the other reflections is observed, which indicates the anisotropic structure of the copper plate produced by rolling.

### 3.3. Etching Behavior

**Figure 8:**
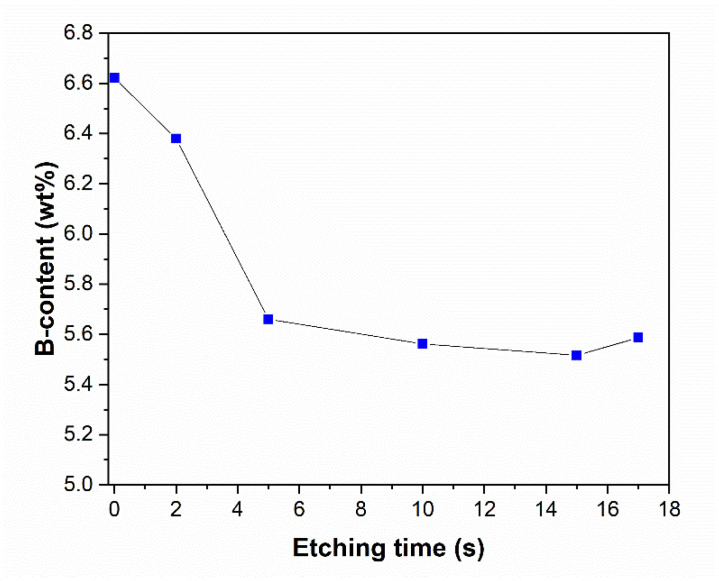
B content of the Ni-B coatings as a function of etching time.

To gain a deeper understanding in the etching mechanism, ICP measurement was used to obtain the B content of the etched Ni-B coatings (Figure 8). As can be seen, the B content of the coatings decreases with increasing etching time, referring to the selective dissolution of B atoms from the coating structure. Interestingly, the opposite was reported in the case of the P-content of the Ni-P coatings [28]. The as-deposited coating has a B content of 6.62 wt%, classified as high-B coating [6,17]. After 5 s of etching, there is approximately 1 wt% drop in the B content, which suggests that B is selectively dissolved from the structure of the coating at the initial stage of etching. By 20 s of etching, the B concentration reaches 5.52–5.59 wt%. The selective dissolution is accompanied by the previously mentioned exponential increase in weight loss over 20 s of etching. These observations suggest a non-uniform surface composition and a time-dependent dissolution mechanism. The higher electrochemical activity of B compared to Ni can promote the selective dissolution of B [38]. During etching, Ni rapidly transforms into Ni(NO_3_)_2_ [39] (in 7 M HNO_3_); meanwhile, the oxidation can leave a thin (Ni and/or B) oxide layer on the surface. At this acid concentration, however, Ni cannot be passivated [40]; thus, the dissolution continues to obtain the observed hollow cylinders (Figure 6b).

## 4. Electrochemical Performance

The effect of acid etching on the electrochemical performance of Ni-B-coated electrodes was investigated by cyclic voltammetry and galvanostatic charge–discharge measurements.

### 4.1. Cyclic Voltammetry

**Figure 9:**
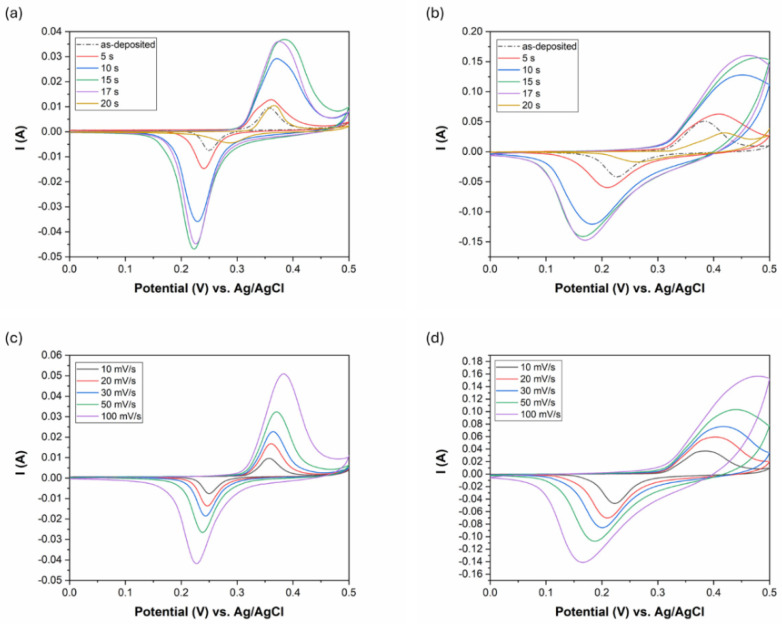
CV results of the Ni-B coatings: (**a**) ν: 10 mV/s with different etching time, (**b**) ν: 100 mV/s with different etching time, (**c**) as-deposited Ni-B with different scan rates, (**d**) Ni-B etched for 15 s with different scan rates, A_electrode_: 5.04 cm^2^.

The potential window of the CV measurement was optimized to the range of 0–0.5 V to fit the extended working range of the etched coatings. The results are shown in Figure 9a,b with different scan rates (10 mV/s and 100 mV/s). The shape of the curves has pseudocapacitive characteristics with two redox (anodic and cathodic) peaks at 0.25 and 0.35 V for the as-deposited coating at a 10 mV/s scanning rate. In alkaline KOH electrolyte, the following reversible faradaic (oxidation reduction) reactions can take place in the case of an etched electroless Ni-B coating [21,41,42]:

(3) $$Ni{\left(OH\right)}_{2}+{OH}^{-}↔NiOOH+H_{2}O+e^{-}$$

(4) $$NiO+{OH}^{-}↔NiOOH+e^{-}$$

(5) $${NiB}_{x}+{\left(6x+2\right)OH}^{-}\to {Ni(OH)}_{2}+{xBO}_{3}^{3-}+{3xH}_{2}O+{(3x+2)e}^{-}$$

Around the anodic peak at 0.35 V, the oxidation of Ni^2+^ to Ni^3+^ takes place, while at around the cathodic peak of 0.25 V, the reverse of this conversion occurs. These reactions require the adsorption and desorption of OH^−^ ions on the surface of the coating. As a zero step, a thin layer of NiO could be formed during the etching process, while a Ni(OH)_2_ layer is formed on the surface of the Ni-B coating when it comes in contact with the KOH electrolyte [22,43,44]:

(6) $${Ni}^{2+}+{2OH}^{-}\to {Ni(OH)}_{2}$$

Since the Ni-B-coated electrodes were soaked in the electrolyte for 24 h, most of the Ni(OH)_2_ layer must be in the form of β-Ni(OH)_2_, which cannot be reduced to Ni due to its passivating effects [45], serving as a durable active material for further redox reactions. The presence of a B-alloying element also contributes significantly to the amount of charge that can be stored on the surface owing to the combined effect of different factors [21].

As the CV curves show non-linear charge storage characteristics, the determination of specific charge storage (Q_S_) from the curves instead of specific capacitance (C_S_) is more appropriate. It is noticeable that the CV curve area increases with etching time up to 15 s. After 17 s, a slight decrease can be observed, while by 20 s, the area decreases back to the CV area of the as-deposited coating. This might indicate that the specific charge storage of the electrodes that correlates with the CV curve area varies as a function of etching time according to a maximum function. The deterioration of the charge storage capability of Ni-B etched for 17 s is possibly due to the reduction in its specific surface area (Figure 4), while after 20 s is due to the loss of active material content on the surface of Cu (Figure 2 and Figure 6a).

The increase in the curve area and the increase in the peak currents (Figure 9a,b) suggest enhanced incorporation of electrolyte ions and surface electrons at the electrode/electrolyte interface owing to the higher specific surface area and interconnected pores of the coating. The symmetry of the anodic and cathodic peaks suggests good reversibility of the redox reactions at 10 mV/s (Figure 9a). However, increasing the scan rate to 100 mV/s (Figure 9b) results in a slight distortion of the anodic peaks in the case of Ni-B etched for 10, 15, and 17 s. This can be attributed to the solution (electrolyte) and pore resistance (diffusion inhibition in the pores), since the current is much higher and this causes a larger voltage drop across the resistances.

The redox peak separation is usually an indicator of the rate of oxidation-reducing reactions. It is found that the peak separation increases with etching time, i.e., with increasing porosity of the coating structure (Figure 9a,b) and with increasing scanning rates (Figure 9c,d). This means that the cathodic peak is shifted toward more negative values while the anodic peak is shifted toward more positive values. The peak separation usually indicates multi-step oxidation/reduction reactions, while its increase refers to decelerated redox reaction rates, which require excess potential to obtain faster ion diffusion and to keep pace with the electronic neutralization of the reactions [29].

**Figure 10:**
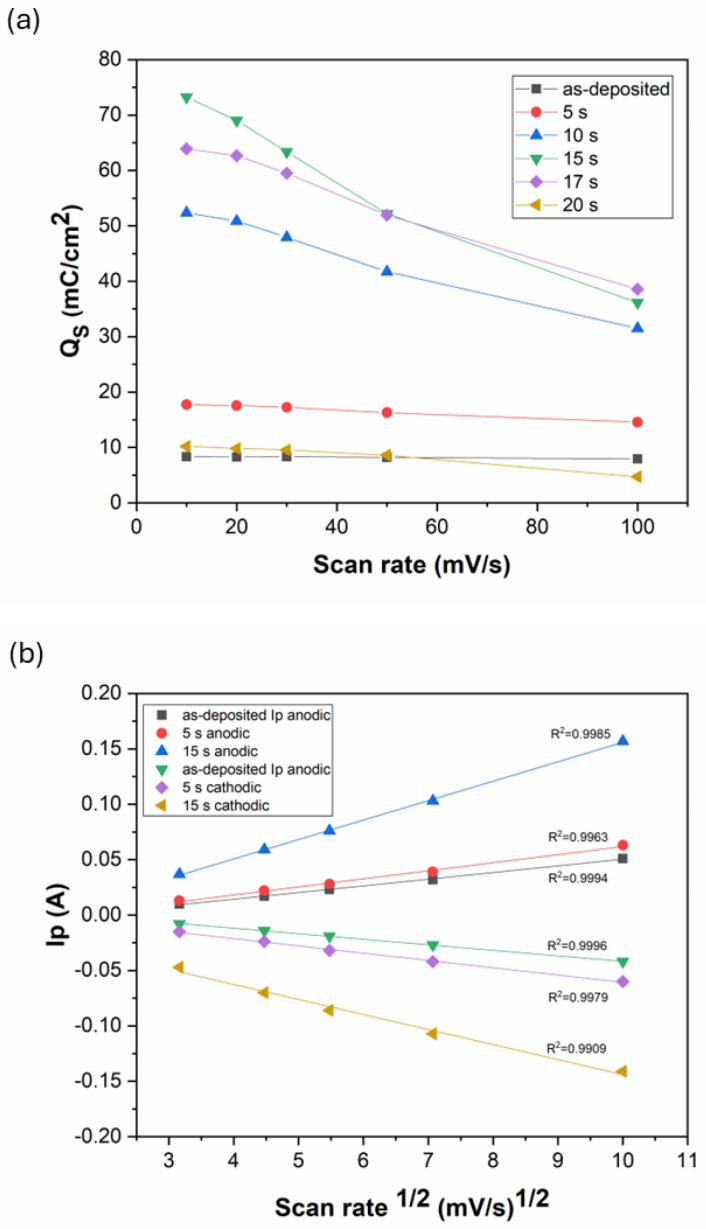
(**a**) Specific charge storage of the Ni-B coatings etched for different times as a function of scan rate; (**b**) anodic and cathodic peak currents as function of the square root of the scan rate.

As seen from Figure 9c,d, the anodic and cathodic peak current rise with the increase in the scan rate. At the same time, the specific charge storage of the coatings decreases with an increasing scan rate (Figure 10a). The level of capacitance decrease (while changing the scan rate from 10 mV/s to 100 mV/s) depends on the specific charge storage capability, as it is ~8% for the as-deposited coating and ~50% for Ni-B etched for 15 s. Faster scan rates usually increase the anodic/cathodic peak currents due to accelerated ionic movement and higher ionic flux within the electrolyte. However, with higher ionic flux, either the time for the reactions to proceed or the diffusion time of the ions to reach the inner pores is not sufficient, thus decreasing the effective surface area and charge storage capacity. In addition to limited time, the diffusion of ions is also affected by the interconnected pore structure. In smaller, narrower pores, ion penetration and flow are limited, thereby reducing the effective surface area of the electrode for energy storage at higher scan rates (Figure 10a) [28,46].

The anodic and cathodic peak currents were found to correlate linearly with the square root of the scan rate, with R^2^ > 0.99 for each Ni-B coating (Figure 10b). This means that the redox reactions of the Ni-B coating are diffusion-limited processes [47]. The $Ni{\left(OH\right)}_{2}↔NiOOH$ transformation was reported to be limited by proton diffusion [45,48]. During discharge, the ${OH}^{-}$ ions move towards the Ni(OH)_2_ surface layer to react with it, and form NiOOH (Equation (3)). Meanwhile, an electron is released and flows towards the external current. However, for this reaction to take place, the mobilization of H^+^ between the Ni(OH)_2_ and NiOOH layers is also needed that proceeds by solid-state diffusion within the solid phases. This internal transport of H^+^ can slow down the whole reaction, especially at high scan rates. Thus, it is believed that the diffusion rate of ${OH}^{-}$ ions in the electrolyte and H^+^ ions in the solid surface layer are responsible for controlling the reaction rates.

### 4.2. Galvanostatic Charge–Discharge (GCD) and Specific Capacitance

**Figure 11:**
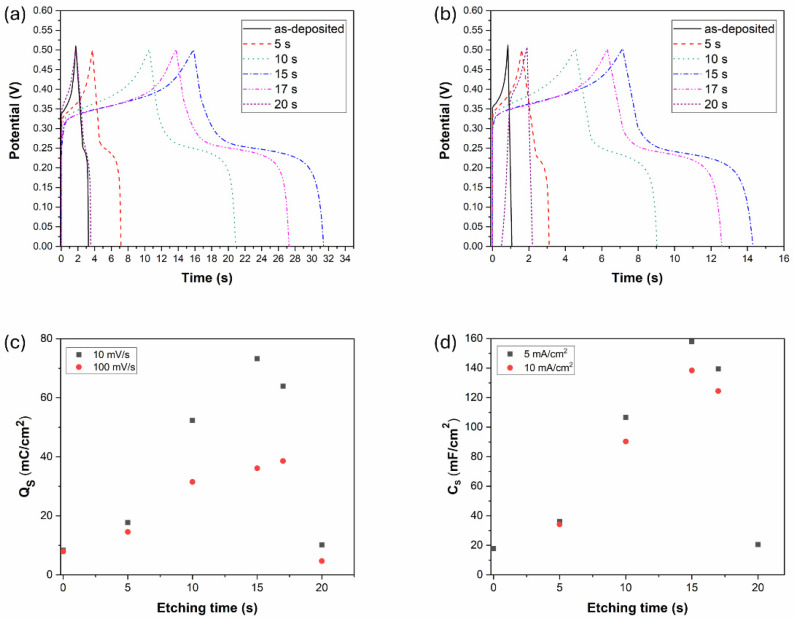
GCD results of the Ni-B coatings with different etching time: (**a**) j: 5 mA/cm^2^, (**b**) j: 10 mA/cm^2^, (**c**) specific charge storage of the Ni-B coatings obtained from the CV results, (**d**) specific capacity of the Ni-B coatings obtained from the GCD results.

The electrochemical behavior of the Ni-B-coated electrodes was further studied by galvanostatic charge–discharge measurement. As can be seen in Figure 11, the coatings show non-linear quasi-symmetric GCD curves with the presence of a plateau, being also an indicator of faradaic reactions [11,49]. The duration of charging/discharging increases with the etching time until 15 s and then decreases back to the performance of the as-deposited coating by 20 s of etching. These results are in correlation with the CV results, as the increasing discharge time refers to higher charge storage capability owing to the porous structure introduced into the coating. At higher current density (10 mA/cm^2^), the charging/discharging time decreases, while the potential drop (IR drop) caused by electrical resistance increases (Figure 11b). It is also seen that Ni-B etched for 20 s became unstable (Figure 11b) during the charging/discharging cycles, which is reflected in the fact that the charge cycle started below 0 V. It was observed that the coating was partially stripped off the surface of Cu (Figure 6); thus, the capacitive behavior was significantly reduced as the charge/discharge obtained a more ohmic (resistance dependent) character, resulting in the voltage at the end of discharge cycle to drop excessively.

The specific charge storage and specific capacities of the Ni-B-coated electrodes can be seen in Figure 11c,d calculated from the CV and GCD results, respectively. The tendency is the same in both cases, increasing to a maximum value at 15 s, and then decreasing back approximately to the capacitance of the as-deposited coating. The highest capacitance of 158 mF/cm^2^ was reached for Ni-B etched for 15 s, at 5 mA/cm^2^ current density: this is more than eight times higher (~778% increment) than the as-deposited state (18 mF/cm^2^) with the original structure with a low degree of porosity. In comparison, an electroless Ni-P coating was reported to achieve a capacitance increment of ~3900% through acid etching [28]. However, the study emphasizes the role of coating mass reduction during etching, which also contributes to the gravimetric capacitance increase. Compared to other pseudocapacitive materials, e.g., MnO_2_ and VS_2_, a capacitance increment of 64–100% was reported as a result of chemical etching. The main identified contributing mechanisms were the large ion-accessible area, and the promotion of kinetics of electrochemical processes provided by etching [50,51]. In the case of EDLC materials, activated carbon was reported to achieve a specific surface area increment of 75% while increasing the capacitance by ~110% via surface treatment (nitric acid fluxing). The treatment introduced an additional pseudocapacitive mechanism to the supercapacitive behavior [52]. Our finding supports the role of specific surface area and pore structure in energy storage capability, as the number of ions and electrons that accumulate and incorporate within the electrode material increases with a higher degree of porosity. Even though the PV depth (i.e., the surface layer depth accessible by electrolytic ions) decreased to ~30% of the as-deposited structure after 15 s of etching (Figure 5b,d), the introduced porous structure and the formed larger cavities located close to each other enable a significant increase in the charge storage capability.

### 4.3. Electrochemical Impedance Spectroscopy

**Figure 12:**
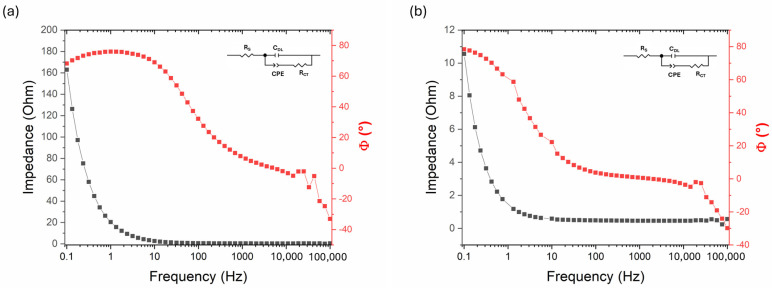
Electrochemical impedance spectroscopy (Bode) plots of (**a**) as-deposited Ni-B and (**b**) Ni-B etched for 15 s.

Electrochemical Impedance Spectroscopy (EIS) measurements were performed on both the as-deposited Ni-B electrode and Ni-B electrode etched for 15 s (Figure 12) at the most characteristic potential, i.e., the potential corresponding to the positive peak of the cyclic voltammograms. The impedance of the as-deposited Ni-B is relatively high (indicating low capacitance), whereas after 15 s of etching it decreases significantly, especially at low frequencies, which is a consequence of the increased capacitance and specific surface area. Even though the relatively simple structure of the spectra does not allow the fitting of a complex model, an equivalent circuit shown in Figure 12 was used.

In addition to the solution resistance (*R*_S_) and the double-layer capacitance (*C*_DL_), a constant phase element represents the pseudocapacitance of the Ni-B layer (*CPE*) in series with a charge transfer resistance (*R*_CT_). The chosen model is rather formal; however, an appropriate fit was obtained (Table 4). The measured double-layer capacitance (*C*_DL_) clearly increased after etching, reflecting the significant enlargement of the electrochemically active surface area. However, the total capacitance determined from galvanostatic charge–discharge measurements was considerably higher, indicating that the majority of the charge storage originates from faradaic (pseudocapacitive) processes, as supported by the presence of a *CPE* in the fitted model. The *R*_CT_ proved to be the most uncertain parameter in both cases; however, the results indicate that the highly porous structure formed by etching did not significantly affect the kinetic control of the electrode. Thus, it can be stated that the charge storage capability is improved mainly capacitively, not kinetically.

### 4.4. Cyclic Life

**Figure 13:**
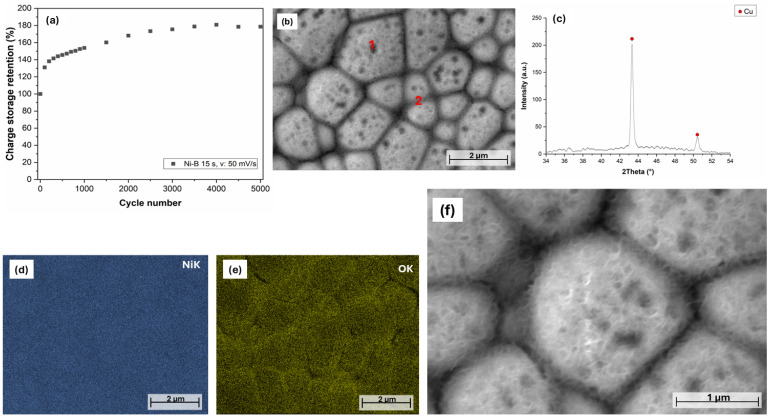
(**a**) Cyclic performance of the Ni-B coating etched for 15 s: v: 50 mV/s, (**b**) PFIB-SEM image of the surface of Ni-B coating etched for 15 s, after 5000 CV cycles, (**c**) XRD diffractogram of Ni-B 15 s after 5000 cycles, (**d**,**e**) elemental map of the surface of the Ni-B coating, (**f**) high-magnification PFIB-SEM image of the surface of the coating.

The cyclic stability of Ni-B etched for 15 s was investigated by CV measurements over 5000 cycles (Figure 12a). Interestingly, the charge storage capability of the coating increases until 4000 cycles, reaching 180% (Figure 13a). The obtained specific value is ~123 mC/cm^2^, which exceeds the areal capacitance reported for highly porous Ni(OH)_2_ nanowall arrays deposited on roughened steel surfaces [53] (by calculating the specific charge storage from the reported capacitance). The rise in charge storage capability/capacitance with the number of cycles is attributed to the fact that pseudocapacitive materials often need to undergo several charge–discharge cycles to be fully activated and provide maximum faradaic capacitance [46,54,55]. The PFIB-SEM images (Figure 13b,f) show the morphological and structural changes of the coating after 5000 CV cycles. These changes are most prominent at the particle boundaries (Figure 13b). The elemental composition of the coating also changed during cycling, reaching a relatively high O content (Table 5), compared to the initial 1.31 wt.% (Table 3). Moreover, O accumulation can be seen at the grain boundaries (Figure 13d,e and Table 5). Higher magnification reveals a very thin, sponge-like structure on the surface, consisting of flat flakes (Figure 13f) covering the entire surface of the coating. To study the thin nanostructured flakes, the coating was measured by XRD in grazing incidence (GI) mode (2° incidence angle). The diffractogram, however, shows no sign of crystalline phases, apart from the reflections of the Cu plate (Figure 13c). Yu et al. [56] observed a capacitance retention of 230% after 2000 cycles for a hierarchical Ni coating deposited on Ni foam. The capacitance increment was attributed to the morphological change of the surface, i.e., the expanding of Ni particles and the formation of Ni(OH)_2_ flakes. As NiO, even when formed by CV processes, is normally crystalline [57], the evolved formations are most likely to be amorphous Ni(OH)_2_ flakes that form an interconnected network, significantly increasing the surface area available for the redox reactions, in line with the reported study. Although the Ni-B-coated electrodes were pretreated by 24 h soaking, the dynamic cycles can further help to open the surface structure (supposedly a few atomic layers); thus, Ni atoms can be oxidized from the deeper parts of the coating as well. Furthermore, the alternation of oxidation reduction reactions during CV cycles can contribute to the formation of a fluffy active layer structure that is easily accessible by the electrolyte ions, similarly to the oxidation of Ni by wide-potential interval CV processes [57].

After 4000 cycles, the charge storage retention slightly decreases and maintains a stable value up to 5000 cycles, which is still much higher (~178%) than the initial value. This confirms the following statements: (1) the coating structure is stable in the long term, with no significant structural degradation; (2) the Ni(OH)_2_/NiOOH transformation of the active layer results in the evolution of a porous Ni(OH)_2_ network, thus further increasing the achievable charge storage capability; and (3) the Ni-B coating of the electrode has good adhesion and mechanical stability on the surface of Cu.

## 5. Conclusions

In this study, the effects of nitric acid etching on the microstructural/morphological changes, alteration in B content, and electrochemical performance of Ni-B-coated Cu plates were extensively studied. It was found that the dissolution of the coating can be described by an exponential function over the investigated period of 20 s, while the B content of the coating decreases by 5 s of dissolution, and then remains constant. The etching introduced a submicron-sized porous structure into the coating, with pores of increasing width and depth with etching time, until transforming into hollow cylindrical structures after 20 s of etching. The electrochemical performance of the Ni-B coatings was investigated with CV, GCD, and EIS measurements. The coatings exhibited reversible pseudocapacitive behavior associated with diffusion-limited redox reactions. An optimal etching time of 15 s was identified, leading to more than an eightfold increase in specific capacitance, reaching 158 mF/cm^2^. It was found that etching primarily enhances the capacitive charge storage properties of the electrode.

The cyclability of the coating showed an outstanding charge storage retention of 180% after 4000 cycles, while 178% was maintained up to 5000 cycles. The increment is attributed to the evolution of a sponge-like porous active layer network consisting of flat Ni(OH)_2_ flakes. The achieved charge storage capability of 123 mC/cm^2^ is attributed to the combination of chemical etching and repeated CV cycles, confirming both the electrochemical and mechanical stability and reliability of the prepared electrode.

## Figures and Tables

**Table 1:** Chemical composition of Cu plates determined by XRF.

Element	Cu	Si	Al	Zn	P	Ni
**Composition (wt.%)**	99.61	0.19	0.09	0.07	0.02	0.01

**Table 2:** Electroless Ni-B bath composition and operating conditions.

**Bath Composition**	**Concentration**
NiCl_2_ (g/L)	40
EDA (g/L)	90
NaOH (g/L)	90
NaBH_4_ (g/L)	0.8
Thiourea (mg/L)	1
**Conditions**	
pH	>13
T (°C)	80
Deposition time	60 min
Bath volume	50 mL

**Table 3:** Elemental composition of the as-deposited Ni-B coating, obtained by EDS.

Element	C	O	Cu	Ni
**Composition (wt.%)**	3.68	1.31	2.13	92.88

**Table 4:** Fitting parameters in equivalent circuits of Ni-B coatings.

**Element**	**As-Deposited Ni-B**		
	**Value**	**Error%**	**Chi-Squared**
R_S_ (Ohm)	0.4479	1.34	0.0015667
C_DL_ (F)	0.0027	13.38	
CPE (F·s^P−1^)	0.0072	5.16	
P (-)	0.8187	1.41	
R_CT_ (Ohm)	0.3202	41.61	
	**Ni-B etched for 15 s**		
R_S_ (Ohm)	0.4696	0.34	0.00020966
C_DL_ (F)	0.0479	5.21	
CPE (F·s^P−1^)	0.1047	2.41	
P (-)	0.8640	0.53	
R_CT_ (Ohm)	0.2215	10.33	

**Table 5:** Elemental composition of Ni-B etched for 15 s after 5000 CV cycles at the measurement points indicated in Figure 12b.

Element		O	K	Ni	Cu
**Composition (wt.%)**	Point 1	5.42	0.22	92.12	2.24
	Point 2	11.71	0.20	83.83	4.25

## Data Availability

The original contributions presented in this study are included in the article. Further inquiries can be directed to the corresponding author.
